# Neuronal NMNAT2 Overexpression Does Not Achieve Significant Neuroprotection in Experimental Autoimmune Encephalomyelitis/Optic Neuritis

**DOI:** 10.3389/fncel.2021.754651

**Published:** 2021-10-11

**Authors:** Pingting Liu, Haoliang Huang, Fang Fang, Liang Liu, Liang Li, Xue Feng, Wei Chen, Roopa Dalal, Yang Sun, Yang Hu

**Affiliations:** ^1^Department of Ophthalmology, Stanford University School of Medicine, Palo Alto, CA, United States; ^2^Department of Ophthalmology, The Second Xiangya Hospital, Central South University, Changsha, China

**Keywords:** optic neuritis, neuroprotection, multiple sclerosis, neurodegeneration, NMNAT2

## Abstract

Optic neuritis, inflammation, and demyelination of the optic nerve (ON), is one of the most common clinical manifestations of multiple sclerosis; affected patients suffer persistent visual symptoms due to ON degeneration and secondary retinal ganglion cell (RGC) death. The mouse experimental autoimmune encephalomyelitis (EAE) model replicates optic neuritis and significant RGC soma and axon loss. Nicotinamide mononucleotide adenylyltransferases (NMNATs) are NAD^+^-synthetic enzymes that have been shown to be essential for axon integrity, activation of which significantly delays axonal Wallerian degeneration. NMNAT2, which is enriched in axons, has been proposed as a promising therapeutic target for axon injury-induced neurodegeneration. We therefore investigated whether activation of NMNAT2 can be used as a gene therapy strategy for neuroprotection in EAE/optic neuritis. To avoid the confounding effects in inflammatory cells, which play important roles in EAE initiation and progression, we used an RGC-specific promoter to drive the expression of the long half-life NMNAT2 mutant in mouse RGCs *in vivo*. However, optical coherence tomography *in vivo* retina imaging did not reveal significant protection of the ganglion cell complex, and visual function assays, pattern electroretinography, and optokinetic response also showed no improvement in mice with NMNAT2 overexpression. Postmortem histological analysis of retina wholemounts and semithin sections of ON confirmed the *in vivo* results: NMNAT2 activation in RGCs does not provide significant neuroprotection of RGCs in EAE/optic neuritis. Our studies suggest that a different degenerative mechanism than Wallerian degeneration is involved in autoimmune inflammatory axonopathy and that NMNAT2 may not be a major contributor to this mechanism.

## Introduction

Optic neuritis, inflammation, and demyelination of the optic nerve (ON), is one of the most common clinical manifestations of multiple sclerosis (MS). About 25% of MS patients have optic neuritis as the initial symptom, 70% have it during the course of the disease, and about one-third suffer persistent visual symptoms due to the ON degeneration and secondary retinal ganglion cell (RGC) death (Talman et al., 2010; Toosy et al., 2014; Balcer et al., 2015). The pathology and functional deficits of the retina/ON injury in MS are more clinically apparent and quantifiable than those due to damage elsewhere in the central nervous system (CNS). Therefore, multiple clinical trials have used optic neuritis for testing neuroprotective approaches for MS repair (Aktas et al., 2016). The mouse experimental autoimmune encephalomyelitis (EAE) model replicates many clinical symptoms and pathological signs of MS, including optic neuritis and significant RGC soma and axon loss (Meyer et al., 2001; Shao et al., 2004; Shindler et al., 2006; Steinman and Zamvil, 2006; Shindler et al., 2008; Guo et al., 2011; Quinn et al., 2011; Fonseca-Kelly et al., 2012; Huang et al., 2017). Its accessible structures and clear functional readout make the RGC/ON a highly advantageous model system to test neuroprotectants.

The chimeric mutant protein, slow Wallerian degeneration protein (*Wld*^*S*^), contains the full-length NAD^+^-synthetic enzyme nicotinamide mononucleotide adenylyltransferase 1 (NMNAT1) at its C-terminal region; its N-terminal region is part of the ubiquitin ligase UBE4B, which is required for translocation of *Wld*^*S*^ from the nucleus to the axon (Freeman, 2014). That *Wld*^*S*^ significantly delays Wallerian degeneration (Mack et al., 2001) supports the notion that axonal NAD^+^ production is critical for axon survival (Wang et al., 2005, 2015). The NAD^+^ precursor, vitamin B3, has been consistently shown to prevent glaucoma in aged mice (Williams et al., 2017). Among the three NAD^+^-synthetic enzymes, NMNAT2 is enriched in axons, suggesting that it is responsible for maintaining axonal integrity (Berger et al., 2005; Cahoy et al., 2008). However, NMNAT2 is very labile and rapidly depleted after axotomy (Milde et al., 2013a), which downregulates axonal NAD^+^ (Wang et al., 2005) and induces axon degeneration (Gilley and Coleman, 2010; Gilley et al., 2013, 2019; Di Stefano et al., 2015; Coleman and Hoke, 2020). Therefore, NMNAT2 is a promising therapeutic target for protecting axons undergoing Wallerian degeneration. Indeed, NMNAT2 overexpression delays injury-induced axon degeneration both *in vitro* and *in vivo* (Feng et al., 2010; Yan et al., 2010; Ali et al., 2016) and alleviates neurodegeneration in the P301L mouse model of tauopathy (Ljungberg et al., 2012). These results support NMNAT2 enhancement as an important therapeutic strategy for axonopathies.

Because axonal Wallerian degeneration is a major component of early axonal pathology in MS (Dziedzic et al., 2010), we reasoned that overexpression of NMNAT2 in RGCs might achieve neuroprotection in EAE/optic neuritis. We therefore tested an Adeno-associated virus (AAV)-mediated gene therapy strategy (Wang et al., 2020) in the mouse EAE/optic neuritis model and specifically expressed long half-life NMNAT2 mutant (NMNAT2Δex6) (Milde et al., 2013b) in the mouse RGCs *in vivo*. However, we found that although the NMNAT2 overexpression itself has no toxicity effect, it provided no significant neuroprotection or function recovery of RGCs and ON, indicating that manipulating NMNAT2 alone is not sufficient to rescue neurodegeneration induced by autoimmune inflammatory axonopathy.

## Materials and Methods

### Animals

C57BL/6J WT female mice (7–9 weeks old) were purchased from Jackson Laboratories (Bar Harbor, ME, United States) and housed in standard cages on a 12-h light–dark cycle. All experimental procedures were performed in compliance with the animal protocols approved by the IACUC at Stanford University School of Medicine.

### Adeno-Associated Virus Production and Intravitreal Injection

The detailed procedure of AAV production has been described previously (Yang et al., 2014, 2016; Miao et al., 2016; Wang et al., 2020). Briefly, the AAV titers were determined by real-time PCR and diluted to 1.5 × 10^12^ vector genome (vg)/ml. For intravitreal injection, mice were anesthetized by xylazine and ketamine based on their body weight (0.01 mg xylazine/g + 0.08 mg ketamine/g). A pulled and polished microcapillary needle was inserted into the peripheral retina just behind the ora serrata. Approximately 2 μl of the vitreous was removed to allow injection of 2 μl of AAV into the vitreous chamber to achieve 3 × 10^9^ vg/retina.

### Experimental Autoimmune Encephalomyelitis Induction and Clinical Scoring

Two weeks after AAV intravitreal injection, EAE/optic neuritis was induced with myelin oligodendrocyte glycoprotein (MOG_3__3__–__5__5_, GenScript, Cas No.163913-87-9) peptide and pertussis toxin (PTX, List Biological Laboratories, Cambell, CA, United States; Cat. No.181) in 9-week-old female mice with a procedure modified from the established protocol (Hasselmann et al., 2017; Huang et al., 2017). Briefly, 225 μg of MOG_3__3__–__5__5_ peptide emulsified with incomplete Freund’s adjuvant (IFA, BD Difco^TM^, 263910) and 2.5 mg/ml mycobacterium tuberculosis (BD Difco^TM^, 231141) were injected subcutaneously for immunization twice at day 0 and day 7, and 500 ng of PTX in PBS (2.5 μg/ml) was intraperitoneally injected twice at day 0 and day 2 to break down the blood–brain barrier. Mice injected with the same volume of Freund’s adjuvant emulsion without MOG_3__3__–__5__5_ were used as sham controls. The behavioral deficits of these mice were assessed daily with a five-point scale: no disease = 0; partial tail paralysis = 0.5; tail paralysis or waddling gait = 1.0; partial tail paralysis and waddling gait = 1.5; tail paralysis and waddling gait = 2.0; partial limb paralysis = 2.5; paralysis of one limb = 3.0; paralysis of one limb and partial paralysis of another = 3.5; paralysis of two limbs = 4.0; moribund state = 4.5; and death = 5.0.

### Immunohistochemistry of Wholemount and Cross Sections of Retina and Longitudinal Optic Nerve Sections

The detailed procedures have been published before (Huang et al., 2017; Zhang et al., 2019; Li et al., 2020; Wang et al., 2020; Fang et al., 2021). Briefly, after perfusion fixation with 4% PFA in PBS, mice eyeballs and ONs were dissected out and post-fixed with 4% PFA for 2 h at room temperature. Sucrose (30%) was then used for cryoprotection of the tissues. Retinas were dissected out for wholemount retina immunostaining. For cross sections of retina or longitudinal sections of ONs, the eyeballs or ONs were embedded in tissue-tek OCT on dry ice for subsequent cryo-section with a Leica cryostat. The primary antibodies used for immunostaining were: anti-RBPMS (custom made at ProSci Inc.), anti-NMNAT2 (Santa Cruz, Cat. No. sc-515206), anti-HA (Roche, 11867423001), anti-Tuj1 (Biolegend, 845502), anti-MBP (Covance, 808401), anti-CD3 (Santa Cruz Biotechnology, sc-18843), and anti-Iba1 (Wako, 019-19741). For quantification of cells in ONs, three to four fields were randomly sampled from each ON by using a Zeiss confocal microscope with × 40 oil immersion objective lens. Positive cells were counted by NIH ImageJ’s cell counter plugin. For quantification of MBP^+^ area in ONs, we calculated the percentage of the MBP^+^ area against the total area. Positive MBP area was identified based on a fluorescence intensity greater than the baseline intensity threshold and the percentage of positive area was measured by NIH ImageJ.

### Total Optic Nerve Protein Preparation and Immunoblotting

Mice were sacrificed by cervical dislocation. ONs were immediately dissected out and rinsed in pre-cooled PBS. Then 80 μl of RIPA buffer (Thermo Fisher Scientific, 89900) with protease and phosphatase inhibitor (Thermo Fisher Scientific, 78441) was added to each sample and incubated on ice for 30 min after homogenization and cleared by centrifugation at 20,000 *g* for 15 min. The protein concentration of the tissue lysate was measured with the BCA protein assay kit (Thermo Fisher Scientific, 23227) following the protocol of the manufacturer. Then 30 μg of protein from each sample was loaded for Western blotting analysis. The primary antibodies used were anti-HA (Roche, 11867423001) and anti-GAPDH (Cell Signaling, Cat. No. 2118).

### Retinal Ganglion Cell Counting

For RGC counting, the wholemount retinas were imaged with the × 20 objective lens of a Keyence fluorescence microscope tiled by 13 × 13 to cover the entire area of the retina. Concentric circle plugin of NIH Image J was used to divide each retina to eight parts by eight circles in order to define the peripheral area (Supplementary Figure 1E). Ten percent of the peripheral area was stereologically sampled with 100 × 100μm counting frames. All surviving RGCs in the sampled areas were manually identified and counted by ImageJ’s cell counter plugin. The investigators who counted the RGCs were masked to the treatment of the samples.

### Optic Nerve Semi-Thin Sections and Quantification of Myelinated Axons

ONs were post-fixed *in situ* with 2% glutaraldehyde and 2% PFA in 0.1 M PBS. Semi-thin (1 μm) cross sections of the ON 2 mm distal to the eye (globe) were collected. After PPD staining, the semi-thin sections of the ON were imaged through a × 100 oil immersion objective of a Keyence bright field microscope tiled by 13 × 13 to cover the entire area of the ON. The stereological sub-sampling plugin AxonCounter of NIH ImageJ was used to sample 5–10% of the entire ON area with 10 × 10 μm counting frames (Koschade et al., 2019). All surviving axons in the sampled areas were manually identified and counted by using ImageJ’s cell counter plugin. The investigators who counted the axons were masked to the treatment of the samples.

### Optic Nerve Ultrathin Cross Sections and Transmission Electron Microscope Imaging and Quantification of Surviving Axons

To prepare ultrathin sections of ON for TEM, one-micron-epoxy sections were examined under light microscope to determine the precise location to cut ultrathin sections. Ultrathin sections (70 nm) were collected onto formvar-coated copper grids and dried overnight. Sections were stained next day with uranyl acetate for 30 min, washed in PBS, and then stained with lead citrate for 7 min. Sections were again washed and dried before observing under TEM. TEM imaging was performed at the Cell Sciences Imaging Facility at the Beckman Center, Stanford University. The cross sections of the entire ON were examined and imaged randomly without overlap at × 4,000 with 11.6 μm × 11.6 μm frames on a JEOL JEM-1400 TEM microscope (JEOL United States, Inc., Peabody, MA, United States). For each ON, 25–45 images were taken to cover the whole area of the ON. Both myelinated and unmyelinated axons were counted for axon density calculation.

### NAD^+^ Measurement in Optic Nerves

The NAD^+^ levels of the ONs were measured according to the protocol of the manufacturer protocol of the NAD^+^/NADH assay kit (Abnova, KA1657). Two weeks after intravitreal injection of AAV2-mSncg-3HA-NMNAT2Δex6 in the left eyes and control AAV2 in the right eyes, mice were sacrificed by cervical dislocation and the ONs were collected gently and quickly. Six mice were used in total, two optic nerves were pooled together as one measure, thus three measures were performed for NMNAT2 overexpression group and control group, respectively. Samples were homogenized in the NAD^+^ extraction buffer and then heated at 60°C for 5 min. The homogenate added with the assay buffer was centrifuged at 14,000 rpm for 5 min to remove cellular debris. After adding reagents to 40 μl of supernatants and standard solutions, the absorbance was determined at 565 nm by TECAN SPARK plate reader (Tecan, Switzerland). The results were normalized to one microgram of protein concentration.

### Spectral-Domain Optical Coherence Tomography Imaging

The detailed procedure has been published previously (Zhang et al., 2019; Li et al., 2020; Fang et al., 2021). Briefly, after anesthetization and pupil dilation, the retina fundus images were captured with the Heidelberg Spectralis SLO/OCT system (Heidelberg Engineering, Germany). The mouse retina was scanned with the ring scan mode centered by the optic nerve head under high-resolution mode (each B-scan consisted of 1,536 A scans). The ganglion cell complex (GCC) includes the retinal nerve fiber layer (RNFL), ganglion cell layer (GCL), and inner plexiform layer (IPL). The average thickness of the GCC around the optic nerve head was measured manually with the aid of Heidelberg software. The investigators who measured the thickness of GCC were masked to the treatment of the samples.

### Pattern Electroretinogram Recording

The detailed procedure has been published previously (Zhang et al., 2019; Li et al., 2020; Fang et al., 2021). Briefly, after anesthetization and pupil dilation, PERG of both eyes was recorded simultaneously with the Miami PERG system (Intelligent Hearing Systems, Miami, FL, United States) according to the instructions of the manufacturer. Two consecutive recordings of 200 traces were averaged to achieve one readout; each trace recorded up to 1,020 ms. The first positive peak in the waveform was designated as P1 and the second negative peak as N2. The amplitude was measured from P1 to N2.

### Optokinetic Tracking Response

The detailed procedure has been published previously (Zhang et al., 2019). Briefly, mice were placed on a platform in the center of four 17-inch LCD computer monitors (Dell, Phoenix, AZ, United States), with a video camera above the platform to capture the movement of the mouse. A rotating cylinder with vertical sine wave grating was computed and projected to the four monitors by OptoMotry software (Cerebral Mechanics Inc., Lethbridge, Alberta, Canada). The sine wave grating, settled at 100% contrast and speed of 12°/s, provides a virtual-reality environment to measure the spatial acuity (cycle/degree) of the left eye when rotated clockwise and the right eye when rotated counterclockwise. The maximum frequency (cycle/degree) that the mouse could perform tracking was identified and recorded by investigators masked to the treatment.

### Statistical Analysis

GraphPad Prism 7 was used to generate graphs and used for statistical analyses. Data are presented as means ± SEM. Student’s *t*-test was used for two groups comparison and one-way ANOVA with *post hoc* test was used for multiple comparisons.

## Results

### Retinal Ganglion Cell-Specific Expression of NMNAT2Δex6 and Increase in NAD^+^ in Optic Nerve

**FIGURE 1:**
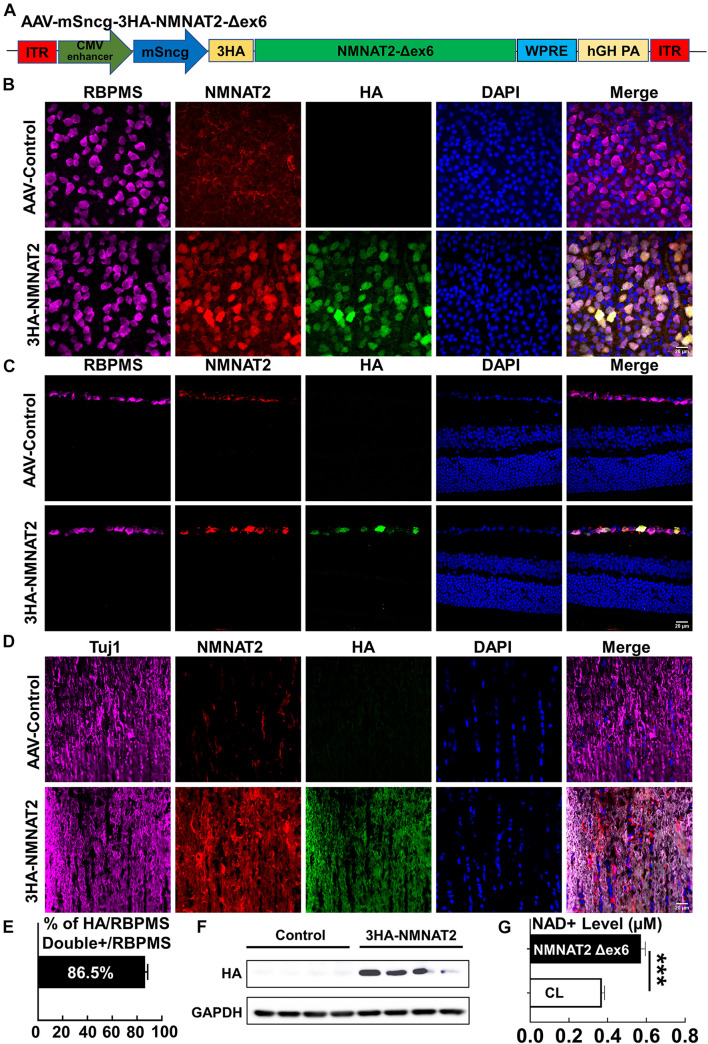
AAV2-mSncg-mediated retinal ganglion cell (RGC)-specific expression of NMNAT2Δex6 *in vivo*. **(A)** Schematic diagram of AAV vector containing mSncg promoter-driven 3HA-NMNAT2Δex6. **(B)** Confocal images of flat-mounted retinas showing RBPMS-positive RGCs and HA-tagged NMNAT2 expression in mice 2 weeks after intravitreal injection of AAV2-mSncg-3HA-NMNAT2Δex6, but not in mice injected with the control AAV2. Scale bar, 20 μm. *n* = 4. **(C)** Confocal images of the retina cross sections showing HA-tagged NMNAT2 expression in RBPMS-positive RGCs but not in the other layers of the retina, 2 weeks after intravitreal injection. Scale bar, 20 μm. *n* = 3. **(D)** Confocal images of optic nerve (ON) longitudinal sections showing HA-tagged NMNAT2 expression in the ONs 2 weeks after intravitreal injection. Scale bar, 20 μm. *n* = 3. **(E)** Quantification of HA/RBPMS-double positive cells in wholemount retinas. Data are presented as means ± SEM, *n* = 4. **(F)** Western blot analysis showing HA-tagged NMNAT2 expression in the ONs 2 weeks after AAV intravitreal injection. *N* = 4. **(G)** NAD^+^ levels in the ONs with or without NMNAT2 overexpression 2 weeks after intravitreal injection. Data are presented as means ± SEM, *n* = 3. ***p < 0.001.

To determine whether the neuronal autonomous effect of NMNAT2 provides RGC and ON protection in EAE/optic neuritis, we first confirmed that NMNAT2 can be expressed in mouse RGCs *in vivo* (Figure 1). NMNAT2Δex6 (soluble forms of NMNAT2, lacking of exon6) is more stable and has greater axon protective capacity than wild-type NMNAT2 (Milde et al., 2013b). Therefore, we drove HA-tagged NMNAT2Δex6 with the RGC-specific mSncg promoter (Wang et al., 2020) in AAV2 vector (Figure 1A). Intravitreal AAV injection achieved RGC-specific expression of HA-NMNAT2Δex6 in the RGC somata in the retina and axons in ON, examined at 2 weeks after AAV injection (Figures 1B–D). Consistent with our previous report (Wang et al., 2020), about 86% RGCs expressed HA-NMNAT2 (Figures 1B,E). Importantly, biochemical assays confirmed the overexpression of 3HA-NMNAT2Δex6 and increase of the NAD^+^ level in the ON 2 weeks after the AAV injection (Figures 1F,G). In summary, we established RGC-specific upregulation of NMNAT2, which enabled us to evaluate the autonomous effect of neuronal NMNAT2 modulation on EAE/optic neuritis neuroprotection.

### Nicotinamide Mononucleotide Adenylyltransferase 2 Overexpression Exerts no Detectable Toxicity on Naïve Mouse Retinal Ganglion Cells

A previous study reported that the exogenous NMNAT2 is toxic to primary cortical neurons (Mayer et al., 2010), and a recent study showed that overexpression of NMNAT2 in the CA1 area *in vivo* enhanced seizure susceptibility and caused neuronal loss (Wu et al., 2021). Therefore, we next investigated the effect of NMNAT2Δex6 overexpression on the viability and visual function of the naïve RGCs. To determine the visual function of living animals, we measured the general electrophysiological activities of the RGCs in response to pattern visual stimuli by PERG (Chou et al., 2014; Porciatti, 2015) and visual acuity by the OKR (Prusky et al., 2004; Douglas et al., 2005). Both procedures are established in our lab (Zhang et al., 2019; Li et al., 2020; Fang et al., 2021). Compared with the contralateral control eyes injected with AAV2 vectors expressing AAV2 capsid itself, there were no significant changes in the P1–N2 amplitude or visual acuity in the NMNAT2-overexpressed eyes 10 weeks after AAV intravitreal injection (Supplementary Figures 1A,B). Consistent with these analyses of the RGC function, there was no significant difference in the RGC morphology between the control and NMNAT2 overexpression eyes: thickness of ganglion cell complex (GCC) in living animals was comparable (Supplementary Figures 1C,D), and histological analysis of post-mortem retina and ON consistently showed no significant RGC somata loss or axon degeneration 10 weeks after AAV injection (Supplementary Figures 1E,F). Therefore, RGC-specific expression of NMNAT2 is non-toxic, at least in the experimental window that we tested.

### Retinal Ganglion Cell-Specific NMNAT2Δex6 Overexpression Does Not Affect Inflammation or Demyelination of Optic Nerve in Experimental Autoimmune Encephalomyelitis

**FIGURE 2:**
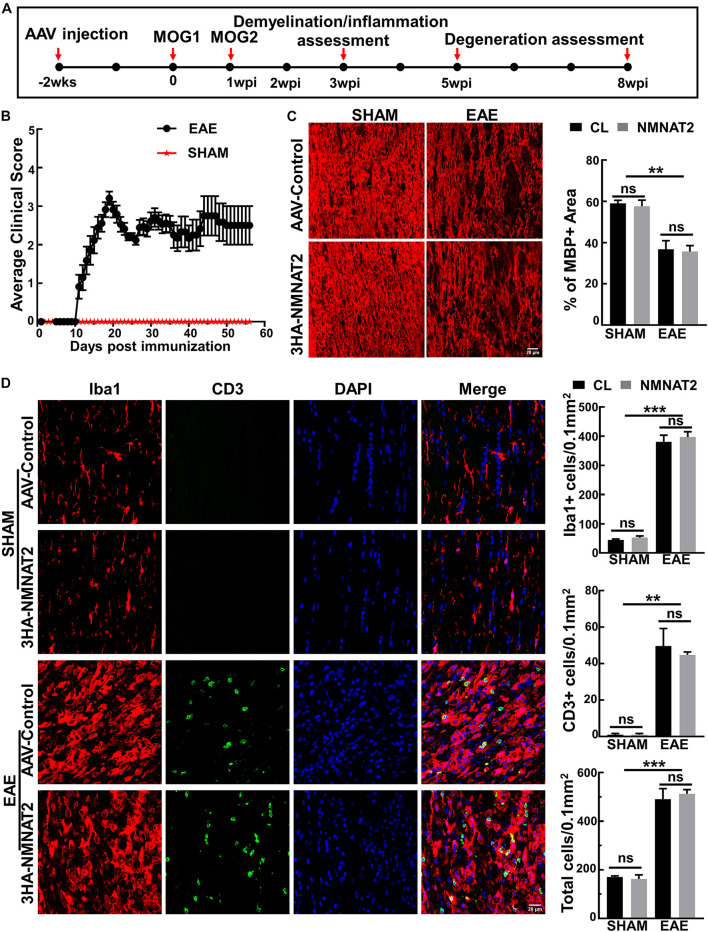
NMNAT2Δex6 overexpression does not reduce inflammation or demyelination of the ON in experimental autoimmune encephalomyelitis (EAE)/optic neuritis. **(A)** Timeline of experimental design showing AAV injection at 2 weeks before first myelin oligodendrocyte glycoprotein (MOG) immunization, second MOG immunization at 1 week post first immunization (1 wpi), inflammation assessment at 3 wpi, and degeneration analysis at 5 and 8 w pi. **(B)** Clinical scores of MOG immunized EAE mice and sham control mice at different time points (days post immunization). Data are presented as means ± SEM. EAE group, *n* = 17; sham group, *n* = 3. **(C)** MBP immunostaining of ON longitudinal sections showing comparable ON myelin basic protein (MBP) decreasing/demyelination in the eyes injected with AAV2-3HA-NMNAT2Δex6 and contralateral control eyes injected with control AAVs at 3 wpi; while there is no ON demyelination in the sham group. Scale bar, 20 μm. Quantification of the percentage of MBP^+^ area to the whole ON area were shown as means ± SEM, *n* = 3. ns, no significance; **p < 0.01, one-way ANOVA with Tukey’s multiple comparisons test. **(D)** Confocal images of the ON longitudinal sections showing significantly increased inflammatory cell infiltration in the EAE mice at 3 wpi with or without NMNAT2 overexpression, but not in the sham control mice. Scale bar, 20 μm. Quantification of Iba1^+^, CD3^+^, and DAPI^+^ total cells in the ONs: Data are presented as means ± SEM, *n* = 3; ns, no significance; **p < 0.01, ***p < 0.001, one-way ANOVA with Tukey’s multiple comparisons test.

Two weeks after intravitreal injection of AAVs, we induced EAE in female mice with myelin oligodendrocyte glycoprotein (MOG_3__3__–__5__5_) peptide and pertussis toxin (PTX) according to the established protocol (Hasselmann et al., 2017; Huang et al., 2017). We performed histological studies of the ON inflammation and demyelination at 3 weeks post MOG immunization (3 wpi) (Figure 2A). Symptoms of weakness began to develop around day 11 after MOG immunization, peaked around day 19, and plateaued for the next several weeks (Figure 2B). The sham group of mice injected with the same reagent mixture except replacing MOG_3__3__–__5__5_ with PBS showed no signs of neurological disease during the experimental course. ON demyelination and optic neuritis were obvious at 3 wpi, evidenced by significant loss of myelin basic protein (MBP) (Figure 2C) and infiltration of inflammatory T-cells and macrophage (Figure 2D). These findings in the EAE mice, but not in the sham mice, were consistent with the time course of EAE/optic neuritis observed in our previous study of this model (Huang et al., 2017). We found no immune cell infiltration in the retinas of these mice (Supplementary Figure 2). Importantly, RGC-specific NMNAT2 expression did not affect the demyelination or inflammation in the ONs of the EAE mice (Figures 2C,D).

### Longitudinal Morphology and Visual Function Studies in Mice With Experimental Autoimmune Encephalomyelitis/Optic Neuritis Show No Evidence of Neuroprotection From NMNAT2Δex6 Overexpression

**FIGURE 3:**
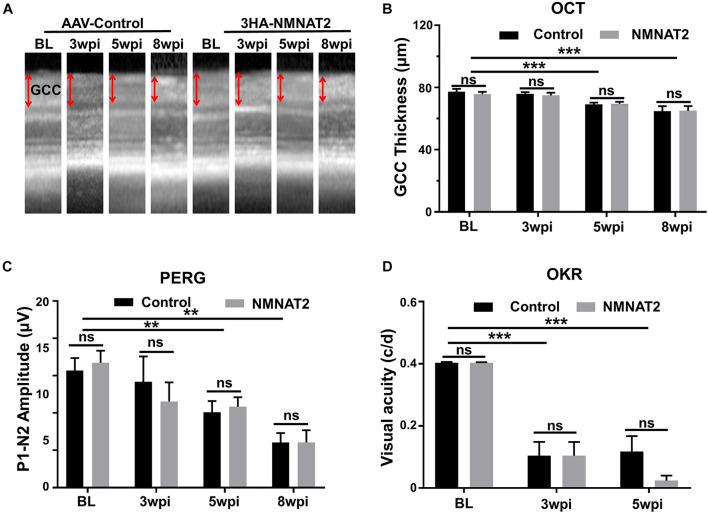
Longitudinal *in vivo* studies show that nicotinamide mononucleotide adenylyltransferases 2 (NMNAT2) provides neither morphological nor visual functional protection in EAE/optic neuritis. **(A)** Representative optical coherence tomography (OCT) images of the mouse retina at different time points after immunization in the EAE mice. GCC, ganglion cell complex, including retinal nerve fiber layer (RNFL), ganglion cell layer (GCL), and inner plexiform layer (IPL); indicated as double end arrows. BL, baseline, before immunization. **(B)** Quantification of GCC thickness measured by OCT at different time points after immunization in the EAE mice. *n* = 16 at BL to 5 wpi; *n* = 6 at 8 wpi. Data are presented as means ± SEM, ***p < 0.001, one-way ANOVA with Tukey’s multiple comparisons test. Comparison of NMNAT2-injected eyes and contralateral control eyes injected with control AAVs, ns, no significance, paired two-tailed *t*-test. **(C)** Quantification of P1–N2 amplitude of PERG at different time points. *n* = 11 at BL to 5 wpi; *n* = 6 at 8 wpi. Data are presented as means ± SEM, ***p < 0.001, one-way ANOVA with Tukey’s multiple comparisons test. Comparison of NMNAT2-injected eyes and contralateral control eyes injected with control AAVs, ns, no significance, paired two-tailed *t*-test. **(D)** Visual acuity measured by optokinetic tracking response (OKR) at different time points. c/d, cycle/degree. *n* = 11 at BL to 5 wpi. Data are presented as means ± SEM, **p < 0.01, one-way ANOVA with Tukey’s multiple comparisons test. Comparison of NMNAT2-injected eyes and contralateral control eyes injected with control AAVs, ns, no significance, paired two-tailed *t*-test.

After confirming the RGC-specific expression of NMNAT2, we investigated its effect on the retina morphology and visual function in mice with EAE/optic neuritis. The GCC thickness measured by OCT in living animals was relatively normal at 3 wpi but decreased progressively at 5 and 8 wpi (Figures 3A,B), consistent with the previous time course of RGC degeneration described previously in EAE mice (Huang et al., 2017). However, there was no significant difference between eyes with NMNAT2 overexpression and contralateral control eyes injected with control AAVs (Figures 3A,B), suggesting no neuroprotection by NMNAT2 overexpression. PERG and OKR measured at different time points after immunization also revealed no significant difference in the P1–N2 amplitude (PERG) or visual acuity (OKR) between NMNAT2 overexpressing eyes and contralateral control eyes, despite the obvious visual deficits in the EAE mice at 3–8 wpi (Figures 3C,D). We sacrificed half of the animals at 5 wpi for histological studies and saved the other animals until 8 wpi. We want to note that the severe paralysis of the EAE mice at 8 wpi precludes reliable measurement with OKR, which relies on body movement in response to visual stimuli. At 5 wpi, the mean visual acuity of NMNAT2 overexpressing eyes (0.024 ± 0.05) was even lower than that of contralateral control eyes (0.118 ± 0.17); however, the difference was not statistically significant (*p*-value, 0.098). Taken together, the RGC-specific NMNAT2Δex6 overexpression did not protect visual function in the EAE mice.

### Retinal Ganglion Cell-Specific NMNAT2Δex6 Overexpression Does Not Promote Retinal Ganglion Cell Soma or Axon Survival in Experimental Autoimmune Encephalomyelitis/Optic Neuritis

**FIGURE 4:**
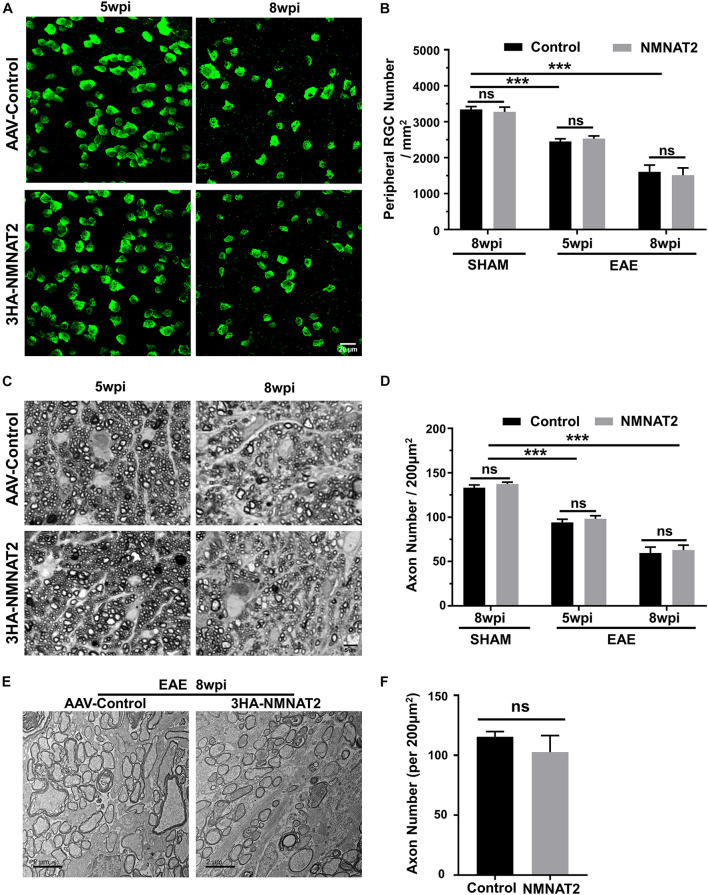
Histological studies confirmed no neuroprotection of EAE RGCs or ON by NMNAT2 overexpression. **(A)** Confocal images of peripheral flat-mounted retinas showing surviving RBPMS-positive (green) RGCs at 5 and 8 wpi in the EAE mice. Scale bar, 20 μm. **(B)** Quantification of surviving RGCs in EAE and sham mice at different time points after immunization. *n* = 10 at 5 wpi; *n* = 6 at 8 wpi; *n* = 3 of the sham mice. Data are presented as means ± SEM, ***p < 0.001, one-way ANOVA with Tukey’s multiple comparisons test. Comparison of NMNAT2-injected eyes and contralateral control eyes injected with control AAVs, ns, no significance, paired two-tailed *t*-test. **(C)** Light microscope images of semi-thin transverse sections of the ON with PPD staining. Scale bar, 5 μm. **(D)** Quantification of surviving axons in the EAE ONs at different time points. *n* = 10 at 5 wpi; *n* = 6 at 8 wpi; *n* = 3 of the sham mice. Data are presented as means ± SEM, ***p < 0.001, one-way ANOVA with Tukey’s multiple comparisons test. Comparison of NMNAT2-injected eyes and contralateral control eyes injected with control AAVs, ns, no significance, paired two-tailed *t*-test. **(E)** Representative TEM images of the ON transverse section at 8 wpi in the EAE mice. **(F)** Quantification of the surviving axons imaged by TEM showed no significant difference between the ONs of NMNAT2-overexpressing eyes and the contralateral control eyes. *n* = 3. Data are presented as means ± SEM, comparison of NMNAT2-injected eyes and contralateral control eyes injected with control AAVs, ns, no significance, paired two-tailed *t*-test.

Quantification of the surviving RGC somata in wholemount retinas (Figures 4A,B) and axons in the ON semi-thin and ultrathin cross-sections images revealed significant RGC soma and axon loss at 5 and 8 wpi (Figures 4C–F). However, RGC-specific NMNAT2Δex6 expression alleviated neither RGC soma nor axon degeneration in mice with EAE/optic neuritis. Taken together, the present results showed that NMNAT2 overexpression in RGCs does not achieve neuroprotection or preserve visual function in EAE/optic neuritis.

## Discussion

In this study, we directly addressed whether neuronal NMNAT2 overexpression rescues neurodegeneration induced by autoimmunity and inflammation. The mouse EAE model demonstrated significant inflammation and demyelination in the ON and associated RGC degeneration and visual functional deficits. However, RGC-specific overexpression of the long half-life NMNAT2 mutant and increased NAD^+^ failed to protect the RGCs or preserve visual function in EAE, indicating that NMNAT2 plays a less important role in autoimmune inflammatory neurodegeneration than in degeneration due to other causes. Consistently, we did not detect significant change of NMNAT2 mRNA levels in the EAE RGCs (data not shown). Thus, these results contrast dramatically with the significant neuroprotection effect of NMNAT2 in traumatic axonopathy and tauopathy (Feng et al., 2010; Gilley and Coleman, 2010; Yan et al., 2010; Ljungberg et al., 2012; Gilley et al., 2013, 2019; Milde et al., 2013b; Ali et al., 2016; Coleman and Hoke, 2020). One possible explanation for this disparity is that, although Wallerian degeneration is detectable in MS axonal pathology (Dziedzic et al., 2010), the axonal degeneration has a distinctly different mechanism in EAE/optic neuritis from the Wallerian degeneration in other neurodegenerative diseases. This notion is supported by an earlier study showing that blocking Wallerian degeneration in inflammatory demyelination does not suffice to prevent axonal loss (Singh et al., 2017). Previous reports show that inflammation-induced neurodegeneration of EAE is attenuated in *Wld*^*S*^ mice (Kaneko et al., 2006; Chitnis et al., 2007), in which all the cells express the Wld^*S*^ (UBE4B/NMNAT1) protein. Thus, another possibility is that the neuronal intrinsic NMNAT-NAD^+^ levels may not be as critical for survival as in the other cell populations in EAE. Exon 6 is essential for normal axonal trafficking of NMNAT2 within neurons. Although the deletion of exon 6 increases its stability and delays Wallerian degeneration (Milde et al., 2013b), NMNAT2Δex6 may function or translocate differently to endogenous NMNAT2. Since we did not directly compare the effect of wild type NMNAT2 to that of NMNAT2Δex6 in this study, we cannot exclude the possibility that overexpression of wild type NMNAT2 may have neuroprotection in the EAE mice.

In summary, RGC-specific NMNAT2Δex6 overexpression and increased NAD^+^ can be achieved safely through AAV2 intravitreal injection with the mSncg promoter, but fails to protect the RGC and ON degeneration in a mouse EAE/optic neuritis model, suggesting that autoimmune inflammatory neurodegeneration is due to different mechanisms from degeneration of other causes.

## Data Availability Statement

The datasets generated for this study are available on request to the corresponding author.

## Ethics Statement

The animal study was reviewed and approved by IACUC at Stanford University School of Medicine.

## Author Contributions

YH and PL designed the experiments and prepared the manuscript. PL, HH, FF, LLiu, LLi, XF, WC, and RD performed the experiments and analyzed the data. YS provided the reagent and conducted the discussion. All authors contributed to the article and approved the submitted version.

## Conflict of Interest

The authors declare that the research was conducted in the absence of any commercial or financial relationships that could be construed as a potential conflict of interest.

## Publisher’s Note

All claims expressed in this article are solely those of the authors and do not necessarily represent those of their affiliated organizations, or those of the publisher, the editors and the reviewers. Any product that may be evaluated in this article, or claim that may be made by its manufacturer, is not guaranteed or endorsed by the publisher.

## Supplementary Material

Supplementary Figure 1 No toxicity by NMNAT2 overexpression in naïve mouse retina. **(A)** Left: representative wave forms of PERG 10 weeks after AAV2 intravitreal injection. Right: quantification of P1-N2 amplitude of PERG. *n* = 3. Data are presented as means ± SEM, ns: no significance, paired two-tailed *t*-test of NMNAT2-injected eyes and contralateral control eyes injected with control AAVs. **(B)** Visual acuity measured by OKR 10 weeks after AAV2 intravitreal injection. c/d: cycle/degree. *n* = 3. Data are presented as means ± SEM, ns: no significance, paired two-tailed *t*-test of NMNAT2-injected eyes and contralateral control eyes injected with control AAVs. **(C)** Representative OCT images of mouse retinas in mice before and after AAV intravitreal injection. GCC: ganglion cell complex, including RNFL, GCL, and IPL layers; indicated as double end arrows. BL: baseline, before immunization. **(D)** Quantification of GCC thickness measured by OCT in eyes injected with AAV-NMNAT2 and contralateral eyes injected with control AAVs. *n* = 3. Data are presented as means ± SEM, ns: no significance, paired two-tailed *t*-test of NMNAT2-injected eyes and contralateral control eyes injected with control AAVs. **(E)** Upper panel: representative wholemount retina image with concentric circles drawn by ImageJ and the areas between the two green circles are eligible for peripheral retina RGC counting. Middle panel: peripheral flat-mounted retinas showing surviving RBPMS positive (green) RGCs 10 weeks after AAV2 intravitreal injection. Scale bar, 20 μm. Lower panel: semi-thin transverse sections of ON with PPD staining. Scale bar, 5 μm. **(F)** Quantification of surviving RGCs in wholemount retina and surviving axons in ONs 10 weeks after AAV2 intravitreal injection. *n* = 3. Data are presented as means ± SEM, ns: no significance, paired two-tailed *t*-test of NMNAT2-injected eyes and contralateral control eyes injected with control AAVs.

Supplementary Figure 2 There is no immune cell infiltration in retinas of EAE/optic neuritis mice. Confocal images of retina cross sections showing no inflammatory cell infiltration in EAE and sham mice at 3 wpi with or without NMNAT2 overexpression. Scale bar, 20 μm. *n* = 3.
