# Supplementary figures and images for: Neuronal NMNAT2 Overexpression Does Not Achieve Significant Neuroprotection in Experimental Autoimmune Encephalomyelitis/Optic Neuritis
Source: Front Cell Neurosci. 2021 Oct 11;15:754651. doi: 10.3389/fncel.2021.754651 (PMC8542903; doi:10.3389/fncel.2021.754651)

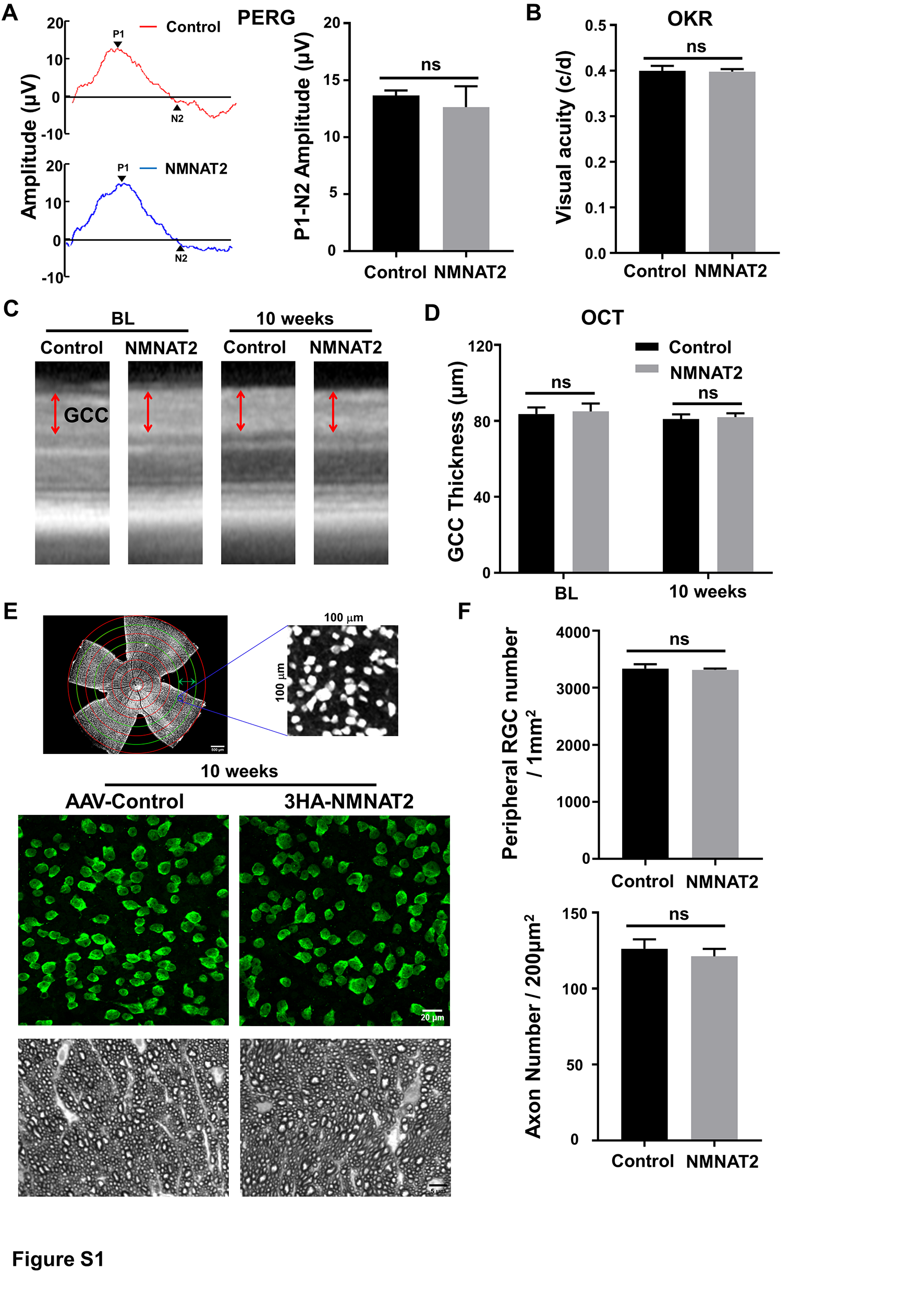

Supplement: Supplementary Figure 1 — No toxicity by NMNAT2 overexpression in naïve mouse retina. (A) Left: representative wave forms of PERG 10 weeks after AAV2 intravitreal injection. Right: quantification of P1-N2 amplitude of PERG. n = 3. Data are presented as means ± SEM, ns: no significance, paired two-tailed t-test of NMNAT2-injected eyes and contralateral control eyes injected with control AAVs. (B) Visual acuity measured by OKR 10 weeks after AAV2 intravitreal injection. c/d: cycle/degree. n = 3. Data are presented as means ± SEM, ns: no significance, paired two-tailed t-test of NMNAT2-injected eyes and contralateral control eyes injected with control AAVs. (C) Representative OCT images of mouse retinas in mice before and after AAV intravitreal injection. GCC: ganglion cell complex, including RNFL, GCL, and IPL layers; indicated as double end arrows. BL: baseline, before immunization. (D) Quantification of GCC thickness measured by OCT in eyes injected with AAV-NMNAT2 and contralateral eyes injected with control AAVs. n = 3. Data are presented as means ± SEM, ns: no significance, paired two-tailed t-test of NMNAT2-injected eyes and contralateral control eyes injected with control AAVs. (E) Upper panel: representative wholemount retina image with concentric circles drawn by ImageJ and the areas between the two green circles are eligible for peripheral retina RGC counting. Middle panel: peripheral flat-mounted retinas showing surviving RBPMS positive (green) RGCs 10 weeks after AAV2 intravitreal injection. Scale bar, 20 μm. Lower panel: semi-thin transverse sections of ON with PPD staining. Scale bar, 5 μm. (F) Quantification of surviving RGCs in wholemount retina and surviving axons in ONs 10 weeks after AAV2 intravitreal injection. n = 3. Data are presented as means ± SEM, ns: no significance, paired two-tailed t-test of NMNAT2-injected eyes and contralateral control eyes injected with control AAVs. [file Image_1.TIF]

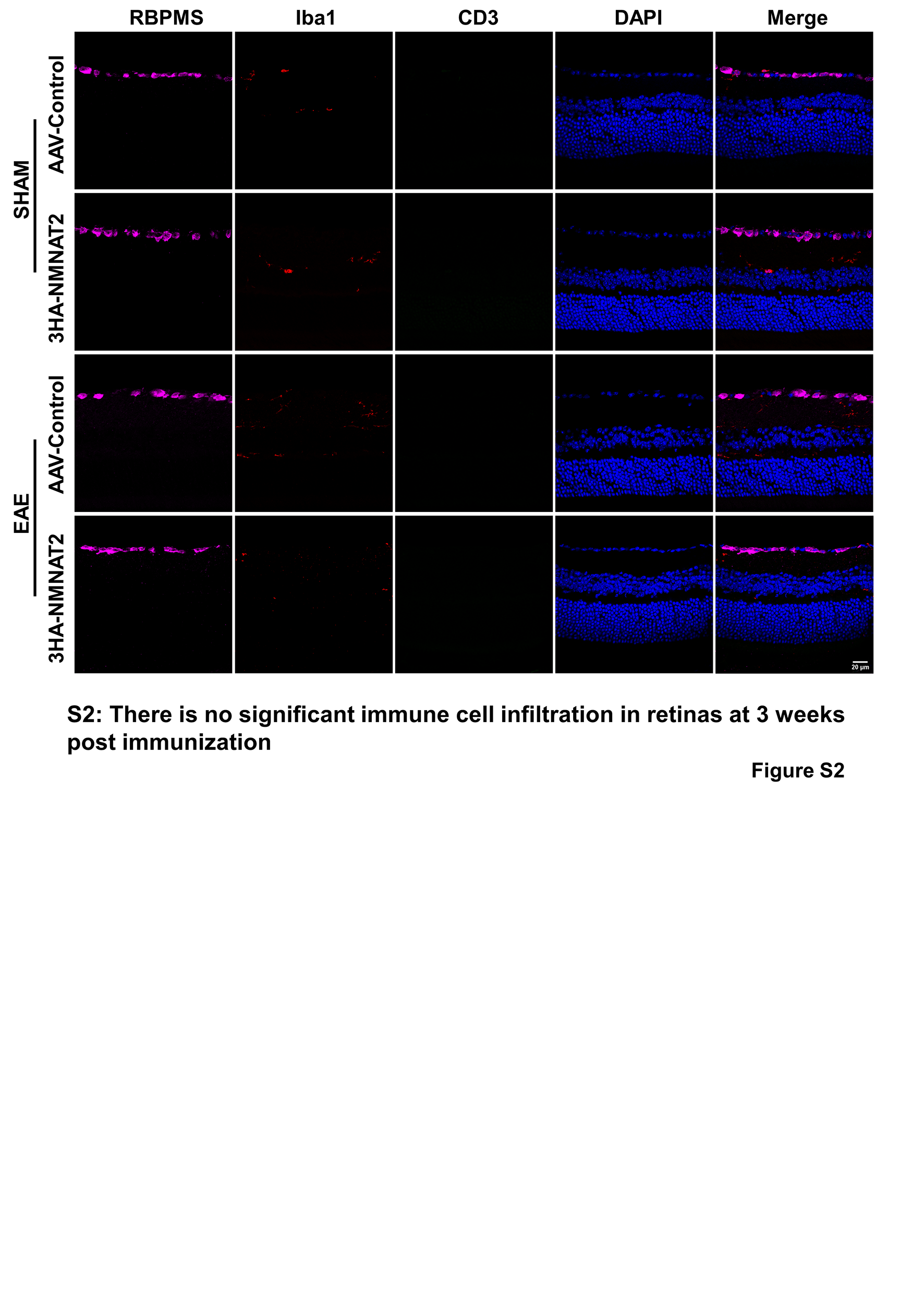

Supplement: Supplementary Figure 2 — There is no immune cell infiltration in retinas of EAE/optic neuritis mice. Confocal images of retina cross sections showing no inflammatory cell infiltration in EAE and sham mice at 3 wpi with or without NMNAT2 overexpression. Scale bar, 20 μm. n = 3. [file Image_2.TIF]
